# Anti-Biofilm Strategies: A Focused Review on Innovative Approaches

**DOI:** 10.3390/microorganisms12040639

**Published:** 2024-03-22

**Authors:** Antonella Iaconis, Laura Maria De Plano, Antonella Caccamo, Domenico Franco, Sabrina Conoci

**Affiliations:** 1 Department of Chemical, Biological, Pharmaceutical and Environmental Sciences (ChiBioFarAm), University of Messina, Viale F. Stagno d’Alcontres 31, 98166 Messina, Italy; iaconis.antonella@outlook.com (A.I.); ldeplano@unime.it (L.M.D.P.); ancaccamo@unime.it (A.C.); 2 Department of Chemistry “Giacomo Ciamician”, Alma Mater Studiorum—University of Bologna, 40126 Bologna, Italy; 3 URT Lab Sens Beyond Nano—CNR-DSFTM, Department of Physical Sciences and Technologies of Matter, University of Messina, Viale F. Stagno D’Alcontres 31, 98166 Messina, Italy

**Keywords:** pathogenic biofilm, anti-biofilm strategies, quorum sensing, quorum quenching, bacterial adhesion, extracellular polymeric substance, phage therapy

## Abstract

Biofilm (BF) can give rise to systemic infections, prolonged hospitalization times, and, in the worst case, death. This review aims to provide an overview of recent strategies for the prevention and destruction of pathogenic BFs. First, the main phases of the life cycle of BF and maturation will be described to identify potential targets for anti-BF approaches. Then, an approach acting on bacterial adhesion, quorum sensing (QS), and the extracellular polymeric substance (EPS) matrix will be introduced and discussed. Finally, bacteriophage-mediated strategies will be presented as innovative approaches against BF inhibition/destruction.

## 1. Introduction

Biofilm (BF) production represents a strategy that bacteria use to survive in adverse conditions and to increase their survival success in the host [1]. Unfavorable conditions can induce bacteria to convert their physiological state from free-floating (planktonic) to sessile cells, acquiring the ability to adhere, grow, and form communities on biotic or abiotic surfaces [2,3]. This physio-metabolic change affects the entire bacterial community by a particular cell–cell communication mechanism, named quorum sensing (QS) [4]. Consequently, the bacterial population coordinates its metabolic activity towards the secretion of an extracellular polymeric substance (EPS), including lipids, polysaccharides, proteins, extracellular nucleic acids (eDNA), and ions [5]. Within this extracellular matrix, bacteria increase their resistance against drying, antimicrobial agents, and the action of the host’s immune system [6]. This finely controlled cooperation often involves different bacterial species, leading to polymicrobial BFs [7,8,9,10]. Bacteria in BFs obtain a common benefit, in terms of growth, virulence, persistence, and the acquisition of antimicrobial resistance (AMR) [11]. The BF extracellular matrix can be considered a hotspot for the diffusion of antibiotic resistance genes, due to the greater frequency and speed of horizontal gene transfer [12]. Therefore, BFs can act as a reservoir of multidrug-resistant (MDR) bacteria, often associated with serious illness and death [11].

The Centers for Disease Control and Prevention estimated that over 2 million infections and 23,000 deaths associated with MDR bacteria occur annually [13]. Among them, six highly virulent and antibiotic MDR bacteria have been included in the ESKAPE (*Enterococcus faecium*, *Staphylococcus aureus*, *Klebsiella pneumoniae*, *Acinetobacter baumannii*, *Pseudomonas aeruginosa*, and *Enterobacter* species) groups [14]. Infections associated with ESKAPE bacteria are generally chronic in the presence of BF and require aggressive therapeutic treatments that subject the patient to serious complications [15]. BF infections can affect the lungs, mainly cystic fibrosis, wounds or medical implants, including orthopedic devices, and intravenous and urinary catheters [16,17]. Because BF infections are very difficult to eradicate, many lines of research are focused on altering the early stages of BF formation [18,19]. Among them, the alteration of QS is emerging as a promising and efficient prevention strategy [20,21,22]. On the other hand, since prevention approaches are not always applicable, several strategies have the EPS matrix as the main target to make pathogenic strains more susceptible to common therapeutic treatments [23,24,25]. Approaches based on bio- and nanotechnology have piqued the interest of numerous research groups due to the possibility of providing greater efficacy to actives and/or obsolete antibiotics [26,27,28,29,30].

This review aims to provide an overview of recent strategies for the prevention and destruction of pathogenic BFs. First, a schematic description of the main phases of the life cycle of BF and maturation will be described, focusing attention on the metabolic pathways involved and the structural components of BFs from Gram-positive and -negative bacteria. Then, recent anti-BF strategies acting on bacterial adhesion to surfaces, QS, and EPS will be introduced and discussed. Ultimately, recent approaches to BF inhibition/destruction by using bacterial viruses will be presented.

## 2. Life Cycle of Biofilm

**Figure 1:**
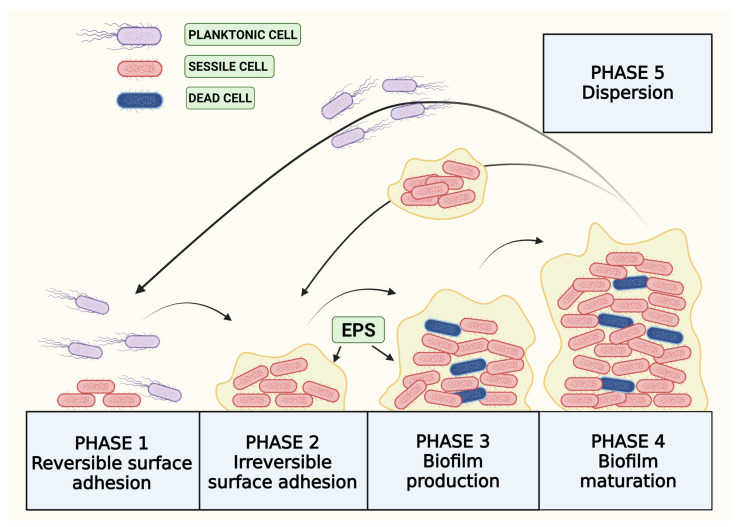
Representation of life cycle of BF formation, from reversible adhesion of bacteria to biofilm dispersion.

The life cycle of BF is a complex phenomenon involving a large number of parameters [31,32]. The main phases of the life cycle of BF are the reversible (i) and irreversible (ii) surface adhesion, BF production (iii) and maturation (iv), and the dispersion (v) of planktonic cells or EPS-included cell aggregates (Figure 1) [33,34].

The life cycle of BF begins with the reversible surface adhesion of planktonic cells (phase 1). Adhesion to biotic or abiotic surfaces is mainly mediated by electrostatic intermolecular interactions, such as acid–base interactions and Van der Waals forces [35,36]. Conformational changes in bacterial surface proteins and an increase in acid–base and hydrophobic interactions progressively maximize the contact with the surface and the removal of interfacial water [37]. In some cases, surface exploration may take place by swarming processes, mediated by Type IV pili or flagella [38]. At this stage (phase 2), bacteria change their physiological state from planktonic to sessile cells by the loss of the superficial appendages and the activation of secondary metabolic pathways [39]. BF production (phase 3) begins with the replication of bacteria by forming mini aggregate microcolonies and the expression of genes related to EPS production and secretion [40,41]. EPS production involves the release of an adhesive matrix which makes cells adhere to each other and allows for the three-dimensional growth of the BF. During BF maturation (phase 4), some cells go towards death and can be used as scaffolds for BF growth. Meanwhile, viable cells import water, nutrients, and other metabolites from the external environment that will be necessary for their survival [42]. At this stage, BFs have the highest resistance against mechanical stresses and adverse environmental factors. The last stage is BF dispersion (phase 5), an active process due to the deterioration and detachment of BF portions with the release of planktonic cells or EPS-included cell aggregates [43]. This phase represents the starting point of a new life cycle of BF formation on other biotic or abiotic surfaces [44].

### 2.1. Reversible Adhesion

Reversible surface adhesion is a crucial step in the BF life cycle [42]. In this stage, bacteria are weakly bound to the surface, mainly due to the absence of EPS, and can explore neighbouring surfaces if deemed more favourable to BF production [36]. On the other hand, the instability of the bacteria makes their remotion easy and, consequently, the blocking of BF production. Motility systems, such as flagella, and the conditioning of the surface through the secretion of polysaccharides are involved in this step [45,46,47]. For example, *S. aureus*, devoid of any flagella, uses Brownian motions to approach surfaces, promoting initial adhesion by using polysaccharide intercellular adhesins (PIAs) and extracellular DNA (eDNA) [48]. Instead, *P. aeruginosa* regulates flagellar motility to promote both adhesion on surfaces and cell–cell adhesion. After reaching the appropriate surface, *P. aeruginosa* minimizes flagellar motility and uses the contraction motility of type IV pili to crawl on the surface and release an exopolysaccharide that promotes surface attachment [49]. Surface characteristics also influence the initial adhesion phase, since they can favour reversible bonds due to hydrophobic and electrostatic interactions [43]. Interactions of bacteria with positive surfaces are generally favoured due to the negative charge of the cell wall [50]. Adhesion strategies differ based on the peculiar characteristics of the cell wall [36]. In Gram-positive bacteria, adhesion is supported by adhesins, binding collagen and fibronectin proteins, and teichoic acids [51]. Bucher et al. identified specific targets, involved in biosynthesis pathways of cell wall components, for hampering BF formation and the anchoring of the extracellular matrix, without affecting planktonic growth [52]. In Gram-negative bacteria, adhesion is favoured by lipopolysaccharides (LPS) in the outer membrane, which makes the bacterial surface highly negative. Abdel-Rhman suggests that LPS in *P. aeruginosa* can stimulate and stabilize BFs [53]. In addition, LPS have a direct stimulatory effect on BFs from other bacterial strains, increasing their virulence [54]. Surfaces of biomedical devices, such as catheters, heart valves, and prostheses, favour the adhesion and subsequent proliferation of pathogenic bacteria, often resulting in chronic infection and health risks to patients [55,56,57].

### 2.2. Extracellular Polymeric Substances (EPSs)

Extracellular polymeric substances (EPSs) play a main role in bacteria survival since they protect bacteria from antibiotics and avoid drug penetration at bactericidal concentrations [58]. The extracellular matrix is an essential component for BF formation and maturation, both in Gram-positive and Gram-negative bacteria [59]. Its components are similar for both bacteria types and include proteins (expressed only during BF formation), polysaccharides, extracellular nucleic acids (eDNA), and some membrane vesicles [60]. BF-associated proteins (Bap), generally present in *S. aureus* [61], have in the central part multiple identical repeats that contain amyloid-like peptide sequences [62]. These proteins promote bacterial adhesion to an abiotic surface by self-assembling into amyloid-like aggregates in response to calcium concentration and low pH values. Gram-negatives also have surface proteins homologous to Bap [47]. Various bacterial species have different numbers of amyloid-like repeats that help in adhesion and immune evasion. Particularly, amyloid-like proteins are present in different species: PSM (phenol-soluble modulins) in *S. aureus*, TasA (translocation-dependent antimicrobial spore component) in *B. subtilis*, CsgA (major curli subunit) in *E. coli*, and Fap in *Pseudomonas* spp. [63,64]. These proteins also act as access channels for nutrients and exhibit surfactant properties that help with dispersal. Amyloid-like proteins are secreted on bacterial surfaces unprocessed, so they require polymerization and processing for final functional activity [47]. Several polysaccharides are also of fundamental importance for BFs. In *S. aureus*, adhesion is enhanced by the surface polysaccharide PNAG (poly-β(1,6)-N-acetyl-D-glucosamine) observed in the formation of immune evasion BF [65]. Meanwhile, in *P. aeruginosa*, exopolysaccharides are secreted including alginate, synthesis locus (Psl), and pellicle (Pel). Alginate may be the major exopolysaccharide responsible for *P. aeruginosa* mucoid formation during chronic infection, while Psl and Pel are more responsible for BF adhesion and maintenance [66]. In *E. coli*, cellulose plays a fundamental role in BF formation; it can also be regulatory and exert the feedback inhibition of BFs [67]. These polysaccharides, in addition to having a role in BF formation, are also important for its maintenance and could be used as targets for the inhibition of BF production [60]. Recently, antibodies capable of binding to the antigenic determinant (epitope) of BF-specific polysaccharides have been tested and developed: for example, human antibodies to *P. aeruginosa* polysaccharide Psl are available and enhance the immune response to BFs [68], while poly-N-acetyl beta-glucosamine has been tested as an antibody against staphylococci [69]. Extracellular DNA, both in Gram-positive and Gram-negative bacteria, confers antibiotic resistance to BFs. DeFrancesco et al. have shown that the use of DNase treatment reduces BFs [70], and therefore this component could be a potential target for future anti-BF therapies. Another important component in BF formation are vesicles which have a fundamental role as hydrophobic surface providers in the transport of EPS constituents, such as eDNA, adhesion-related proteins, and lipids. Membrane vesicles (MVs) are found in Gram-positive bacteria, while outer membrane vesicles (OMVs) in Gram-negative [47]. In *S. aureus*, MVs can carry active β-lactamase, which confers resistance to antibiotics [71]. Otherwise, OMVs produced by Gram-negative transport protein components of the matrix, endotoxins, DNA, and enzymes [72]. A proteomic study indicated that 20% of the protein components of the matrix derive from the OMVs [73]. Potentially, the vesicular components could also be a target for possible anti-BF therapies.

### 2.3. Quorum Sensing in Biofilm Production

**Figure 2:**
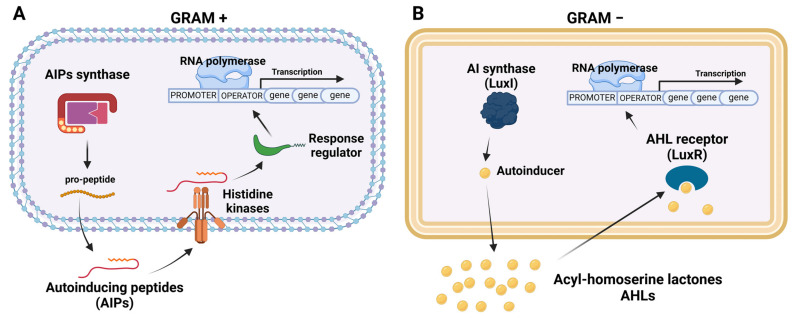
Schematic representation of production and transduction of signals responsible for quorum sensing in Gram-positive (**A**) and Gram-negative (**B**) bacteria.

Quorum sensing (QS) is a bacterial system for regulating gene expression in response to fluctuations in cell population density [4]. Bacteria can communicate and recognize the population density and control gene expression thanks to the release and accumulation of self-inducing extracellular signals, named autoinducers. As the bacterial population increases, autoinducers accumulate above the minimum threshold level: receptors that bind autoinducers trigger signal transduction cascades that lead to population-level changes in gene expression (Figure 2) [74,75]. There are many processes related to QS, including bioluminescence, the secretion of virulence factors, and the sporulation and production of antibiotics [76]. Gram-positive and Gram-negative bacteria have different types of autoinducers, although some systems are present in both types of bacteria [77].

In Gram-positive bacteria, intracellular communication is regulated by autoinducing peptides (AIPs), small post-translationally modified peptides. Several AIPs have been identified, including their structure–activity relationships and cognate receptors [78]. In *S. aureus*, the accessory gene regulator (Agr) is responsible for the regulation and secretion of AIPs (Figure 2A). Four Agr systems regulate the production of many AIPs and each specifically activates its cognate receptor [79]. The Agr system influences protease expression, which facilitates the collapse of mature stage BFs [80] by downregulating the formation of adhesion molecules such as autolysin E (AtIE) [5]. In *S. aureus* planktonic cells, the Agr system is highly active except in the BF state, indicating that the Agr system regulates the shedding of the BF [81]. The primary QS signalling molecule in Gram-negative is N-acylated homoserine-lactone (AHL) [82]. AHL regulates the so-called LuxI/LuxR system. AHL is released extracellularly, and when it accumulates, it enters the cell and binds to its receptor LuxR. Thanks to this binding, LuxR can lead the transcription of the gene responsible for the production of LuxI. LuxI, in turn, is liable for producing AHL itself. LuxI/LuxR regulates virulence factors and BF formation (Figure 2B) [83]. Gram-negative and -positive bacteria widely spread the system mediated by autoinducer-2 (AI-2) [84]. LuxS is a protease, encoded by the *luxS* gene, and regulates the synthesis of AI-2, which in turn can be transported out of the cells. During cell proliferation, the extracellular AI-2 concentration increases, and when reaching a threshold, bacteria sense a critical cell mass. This involves the regulation of QS gene expression, including the formation of BFs and the expression of virulence genes [85]. The two-component system (TCS) is ubiquitous and regulates several bacterial functions including growth, metabolism, pathogenicity, drug resistance, and host recognition [86]. The TCS is involved in planktonic cell morphology and consequently in BF formation [87]. The peculiar feature of this system is the presence of two components: the first component is a protein that acts as a sensor, in this case, histidine protein kinase (HPK), which is found in the inner membrane; the second component is a regulatory protein (RR) [5]. RR activation is responsible for the regulation of genes involved in morphogenesis, virulence factors, and QS [88].

## 3. Anti-BF Strategies Acting on Bacterial Adhesion to Surfaces

**Figure 3:**
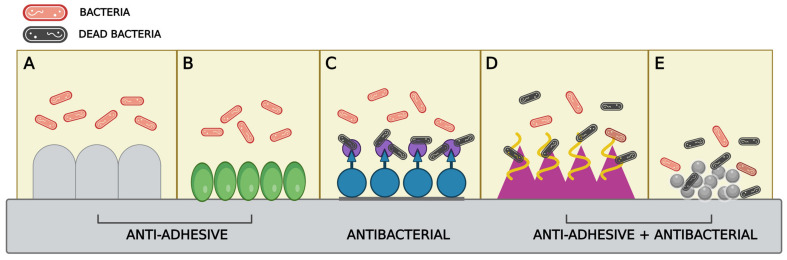
A representation of the possible surface modification strategies for inhibiting initial bacterial adhesion through the modification of the surface of the material used (**A**) or through the use of molecules or polymers that prevent bacteria from adhering to the surface (**B**). Antibacterial action may be facilitated by the presence of molecules capable of killing bacteria (**C**). Additionally, the combined approach of surface modifications with polymers (**D**) or the integration of nanoparticles (**E**) can lead to an antiadhesive surface with antibacterial properties.

Surfaces in contact with biological fluids represent a good niche for bacteria adhesion. Surface adhesion is the crucial event for BF formation which can lead to complicated infections, even localized chronic infections, and serious limitations in the function of biomaterials [89]. Several surfaces favour bacterial adhesion, promoting the transition from planktonic to sessile cells and EPS-mediated cell anchoring [36,90]. Surface modulation represents one of the best approaches in preventing bacterial adhesion and an excellent prevention strategy against pathogenic BFs [91]. Surfaces can be directly altered or added with coatings able to make them inhospitable for bacteria [92]. Physical and chemical surface modulations can prevent bacterial adhesion and release lethal substances for microorganisms so that in addition to the anti-BF action, the surface would be able to prevent bacterial proliferation (Figure 3) [89].

### 3.1. Antiadhesive and Antibacterial Surface Modulation

Over the last 20 years, strategies have been developed to combat resistance associated with BFs but also to prevent microorganisms from being able to produce it [93]. Modulating a surface capable of preventing the first stages of BF formation seems to be a good anti-BF strategy. Zwitterionic materials have antifouling properties and have been widely used to construct antifouling surfaces for medical devices, biosensors, and marine coating applications [94]. Zwitterionic materials prevent bacterial cells from adhering thanks to their electric neutrality with equivalent positive- and negative-charged groups. They are also able to prevent protein attachment and bacterial colonization as their chemical properties allow them the binding of water molecules [95]. Silver-coated surfaces have been extensively studied due to their antimicrobial properties [96,97]. Lemire et al. have demonstrated that surfaces coated with silver oxynitrate have also successfully eliminated multispecies BFs [98]. Medical devices coated with aryl rhodanines specifically inhibit the early stages of BF development in Gram-positive strains, despite not having antibacterial activity [99]. The antiadhesive materials prevent the first surface adhesion, leading to the inhibition of the formation of BFs, in short-term installations as they cannot kill bacteria [100]. Xiang et al. used poly (carboxybetaine-co-dopamine methacrylamide) copolymer (PCBDA) to immobilize silver nanoparticles on cotton gauze resulting in an effective method not only to inhibit BF formation but also to accelerate healing processes [101]. Instead, a new strategy to inhibit the formation of BFs on surfaces could be represented by bacteriocin, a proteinaceous toxin produced by bacteria to inhibit the growth of similar bacterial strains [102]. Indeed, a bacteriocin derived from *Lactobacillus sakei* was able to destroy the BF of *Listeria monocytogenes* on stainless steel [103]. For example, bacteriocins bovicin HC5 and nisin can vary the microbial cell’s hydrophobicity and modulate the microbial cellular attachment, even at sub-inhibitory concentration [104].

### 3.2. Use of Nanoparticles (NPs) in Surface Modulation

In addition to the development of new materials that prevent microorganisms from adhering and starting all those mechanisms that lead to the formation of BF, nanoparticles (NPs) of various kinds have recently been used and deposited on surfaces, to prevent the formation of BFs [105]. NP-embedded materials have now been shown to inhibit BF formation in *E. coli*, *P. aeruginosa*, *S. aureus*, and *S. epidermidis* [106]. The use of NPs can be substantially divided into two strategies: the first involves the use of NPs, of a lipid or polymeric nature, as drug delivery carriers to activate antibiotics; the second involves the use of metallic NPs which act as an antimicrobial agent [107]. NPs can be used associated with enzymes that can degrade the BF’s adhesive structure [100]. For example, α-amylase, a glycoside hydrolase that catalyses the breaking of 1,4-glycosidic bonds, is also able to hydrolyze carbohydrates present in the BF matrix [108]. Silver NPs associated with amylases can eradicate and inhibit BF formation in different bacterial species [100,109]. Hybrids of α-amylase and zinc oxide in an NP form used to coat urinary catheters in a single-step sonochemical approach allowed for a drastic reduction in the incidence of bacteriuria in rabbit models [110]. Thanks to the combination of silver NPs with modified sulfobetaine in polyester membranes, the formation of BFs in *S. aureus* and *E. coli* was inhibited [111]. Furthermore, NPs could inhibit DNA replication and gene expression, thanks to the ability to attach themselves to the microbial surface allowing it to react with proteins and cellular DNA [112]. The nanoparticles most studied to implement anti-BF strategies are gold (Au), silver (Ag), and zinc (Zn) [113]. AuNPs have no antibacterial activity on their own, except when combined with antibiotics. Ampicillin bound to the surface of AuNPs (AuNPs-AMP) allows for the subversion of the drug resistance in different microorganisms such as *E. coli*, *P. aeruginosa*, and *E. aerogenes* [114]. Thanks to the presence of ampicillin, the AuNPs can enter the bacterial cells allowing for them to carry out their antimicrobial function [115,116]. AgNPs have antimicrobial activities and are also responsible for suppressing microbial resistance in terms of growth [117]. Silver ions (Ag+) have antimicrobial activity as they can bind to the negative part of cell membranes, damaging them and therefore allowing the cytoplasmic contents to escape [118]. Thanks to the presence of LPS, negatively charged, Gram-negative bacteria are more sensitive to the action of AgNPs, unlike Gram-positive bacteria which instead have peptoglycans, positively charged [119]. Regarding the use of ZnNPs, it is widely known that the antimicrobial activity of zinc oxide NPs (ZnONPs) is associated with the production of reactive oxygen species (ROS) that can affect cellular components such as lipids, proteins, and nucleic acids, with consequent cellular structural alterations [120]. The use of NPs, and more generally the modulation of surfaces, represents an excellent way forward in the prevention of bacterial adhesion, hindering the formation of BFs; however, there is a need to mainly improve the characterization of NPs on surfaces but also biocompatibility and the assessment of any toxic effect on humans, animals, and the environment [107]. Applying these strategies to inhibit BF formation appears to be the solution with the best perspective as this type of modulation would not lead to the development and worsening of antibiotic resistance [105].

## 4. Anti-BF Strategies Acting on Quorum Sensing

**Figure 4:**
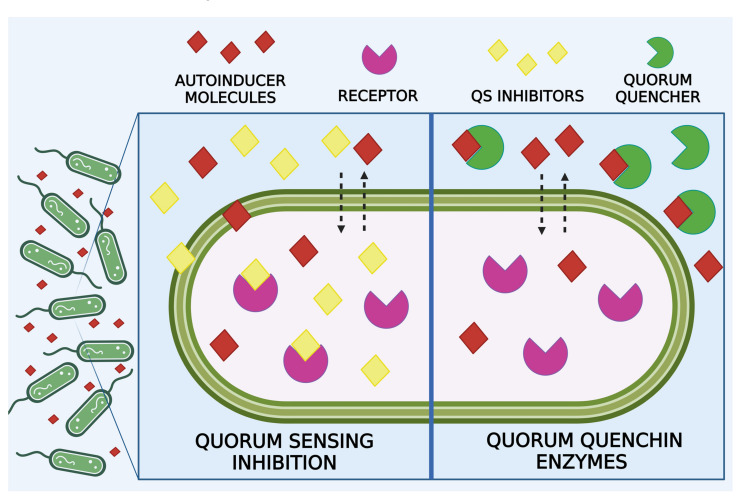
An illustration of bacteria using receptors (purple) to sense the signals (red) produced by nearby bacteria, allowing them to communicate the density of their population. QS can be inhibited thanks to QS inhibitors (yellow) capable of binding receptors. Meanwhile, quorum quenching enzymes (green) can bind to autoinducer molecules.

Quorum sensing (QS) is a mechanism for regulating gene expression based on microbial population density in response to environmental oscillations. QS offers better survival to microbial populations rather than individual cells [4]. In the human health field, because QS is correlated to BFs’ production, several authors are focusing their studies on cell-to-cell communication disruption as an innovative strategy against chronic infections [121]. In-depth studies on what the signals that regulate the formation of BFs are can represent the best strategy against bacterial resistance and immune evasion [5,122,123]. Different molecules, including enzymatic proteins, can degrade bacterial messengers or inactivate specific receptors related to bacterial QS, blocking the metabolic pathways associated with it [6]. Consequently, bacterial cells remain in a dispersed condition and are more susceptible to antimicrobial treatment than BF-residing cells [124]. This phenomenon of the interruption of QS signals is generally referred to as quorum quenching (QQ) (Figure 4) [125].

### 4.1. Quorum Sensing Inhibitors

Several molecules can inhibit or block cell-to-cell communication mediated by QS. Many of these molecules are naturally produced plant metabolites, such as cinnamaldehyde, one of the primary constituents of cinnamon, indicated for its therapeutic potential as an antimicrobial agent against pathogenic BFs [126]. Niu et al. showed that low concentrations of cinnamaldehyde negatively affect two types of QS related to acyl homoserine lactone (AHL) and autoinducer-2 [127]. In the same line of research, Topa et al. combined cinnamaldehyde with colistin to obtain synergistic activity in the inhibition and dispersion of preformed *P. aeruginosa* BF [128]. Again, Li et al. indicated a decrease in the virulence phenotypes of *Aeromomonas hydrophila*, due to QS inhibition and the downregulation of related genes following the addition of cinnamaldehyde [129]. Exploiting nanotechnology advancement, cinnamaldehyde has been loaded on chitosan NPs for anti-BF purposes [130]. Using a similar system, Subhaswaraj et al. obtained a significant anti-quorum sensing activity, mediated by the downregulation of virulence factors such as PAO1 related to QS in *P. aeruginosa* and associated with its BF formation [131]. Moreover, they indicated a significant alteration in the swimming and swarming motility of the same bacterial strain. In the end, Ramasamy et al. conjugated cinnamaldehyde to the surface of gold NPs to obtain a broad spectrum of anti-BF activity against Gram-negative (*E. coli* O157:H7 and *P. aeruginosa*) and -positive (methicillin-sensitive and -resistant *S. aureus*) bacteria [132]. Other compounds able to inhibit QS belong to natural flavonoids, such as baicalein and quercetin, for which a concentration-dependent decrease in violacein production in *C. violaceum* 12,472 and inhibition in pyocyanin production, proteolytic, and elastolytic activities, swarming motility, and BF formation in *P. aeruginosa* PAO1 have been demonstrated, due to quercetin and quercetin-3-O-arabinoside [133]. Paczkowski et al. showed inhibition via the antagonism of the autoinducer-binding receptors, LasR and RhlR, in *P. aeruginosa*. Specifically, a significant reduction in the ability of receptors to bind DNA-encoding QS-regulated promoters, mediated by two hydroxyl groups in the flavone A-ring, has been reported [134]. Similar results were obtained on *A. hydrophila* by citrus flavonoid hesperidin methylchalcone, with a consequent decrease in BF development and virulence factor production [135]. Finally, Pachaiappan et al. investigated the inhibition activity of N-acyl-homoserine lactone mediated by two flavonoids, namely apigenin and acacetin, and three isoflavonoids, namely genistein, daidzein, and biochanin, in *P. aeruginosa* [136]. Flavonoid derivatives have also been evaluated coupled with chitosan delivery systems to enhance the inhibitory activity of BFs from *E. coli* and *P. aeruginosa* [137,138]. Alternative approaches involved the use of silver and gold nanoparticles in flavonoid-based nanohybrids for multidrug-resistant bacteria [139,140]. Another plant metabolite able to prevent the production of QS-controlled virulence factors and related to BF formation is eugenol [141]. Fekrirad et al. indicated that this catechol was able to prevent the production of QS-controlled virulence factors, such as pigment prodigiosin, protease, and hemolysin in *Serratia marcescens* [142]. Specifically, they found that eugenol affected swarming motility, the formation of the microcolony, and extracellular polysaccharide via the downregulation of correlated genes. Also, in this case, approaches combined with other hydrophobic antimicrobial agents (triclosan) and nano- and micro-emulsions, reinforced with silver nanocomposites, have successfully been evaluated against BFs from Gram-positive and -negative bacteria [143,144,145,146]. The activity of QS inhibition has also been documented for some antibiotics, although their main activity is to inhibit growth or act as bactericidal [147,148,149,150,151]. Some authors showed that the macrolide antibiotic azithromycin was able to inhibit QS signal molecules in *P. aeruginosa*, attenuating its virulence [148,149]. Based on this finding, Zeng et al. have investigated the mechanism of action [150]. Since azithromycin acts on ribosomes, it has been evaluated if the transcriptional regulation of representative virulence genes could elicit alternative modes of gene expression mediated by the antibiotic. They suggest a relationship between *lasI* and *rhlI*, for which the first acts as a cell density sensor, while the second functions as a fine-tuning mechanism for the coordination of different QS systems. The ability to reduce the expression of virulence factors in bacterial populations is also documented for other antibiotics. For example, low concentrations of ceftazidime, cefepime, and imipenem caused the significant elimination of the QS signals in *P. aeruginosa* at a concentration 20 times lower than the MIC [151]. The activity is related to a decrease in elastase, protease, pyocyanin, and hemolysin, suggesting a potential use of β-lactam antibiotics as an effective approach for the prevention and treatment of BF infection [151]. To efficiently increase the penetration and retention of antibiotics in BFs, several approaches based on the loading of the antibiotic in small and differently charged NPs have been proposed [152,153]. It is known that a combination of oxazolidinone derivatives compound with β-lactam antibiotics (meropenem trihydrate) can reduce *P. aeruginosa* BFs by inhibiting the virulence factors such as elastase, pyocyanin, rhamnolipid, and protease and bacterial motility [154]. Anti-QS strategies often involve the use of such water-soluble cyclic oligosaccharides to enhance or synergistically act against BFs and other virulence factors [155,156,157].

### 4.2. Metal Nanoparticles as QS Inhibitors

The antibacterial activity associated with metal-based nanoparticles (MeNPs) includes the loss of cell wall and membrane integrity, as well as interference in many metabolic functions essential for bacterial cell viability [158,159]. The main mechanisms include physical damage due to chemical interactions, such as leaching and the dissolution of metal ions, and/or the production of reactive oxygen species (ROS) [160]. Recent evidence has demonstrated that metal NPs are also able to interfere with cell-to-cell communication, acting as QS inhibitors or inductors [161]. Srinivasan et al. have evaluated the anti-QS and anti-BF potential of *Piper betle*-based synthesized silver NPs against *S. marcescens* and *P. mirabilis*. Their results revealed the inhibition of QS-mediated virulence factors, such as prodigiosin, protease, and exopolysaccharides. Specifically, they indicated the downregulation of *fimA*, *fimC*, *flhD*, and *bsmB* genes in *S. marcescens* and *flhB*, *flhD*, and *rsbA* genes in *P. mirabilis*, respectively [162]. Similarly, Shah et al. showed anti-quorum sensing activity in nosocomial pathogen *P. aeruginosa* mediated by photosynthesized silver NPs. They deduced that eugenol-, a phenolic phytochemical, conjugated AgNPs exhibited a considerable binding interaction with QS-associated proteins, such as LasR, LasI, and MvfR [163]. Kumar et al. biosynthesized silver NPs from an aqueous leaf extract of *Koelreuteria paniculata* [164]. Their NPs resulted in the superior inhibition of QS-regulated virulence factors in *P. aeruginosa* PAO1 compared to chemically synthesized AgNPs. Moreover, no effects on cell viability were observed. On the other hand, Saeki et al. evaluated biogenic silver NPs acting on BF formation, the production of virulence factors, and the expression of QS-related genes PAO1 and PA14 in *P. aeruginosa*. [165]. However, their results indicated that exposure to low concentrations of bio-AgNPs could promote the expression of QS genes in *P. aeruginosa*, increasing the production of virulence factors such as elastase, pyocyanin, and BFs [165]. Anti-QS has also been obtained from other MeNPs. Elshaer and Shaaban microbially synthesized gold and selenium with anti-virulent activity against *P. aeruginosa* [166]. NPs inhibited QS-related virulence factors, such as pyocyanin, protease, and elastase, as well as significantly suppressed the expression of QS genes and toxins. Gold NPs from *Capsicum annuum* reduced *P. aeruginosa* and *S. marcescens* BFs, probably by inhibiting QS signals and blocking regulatory proteins [167]. Again, Gómez-Gómez et al. asserted the alteration of the QS signalling system mediated by selenium and tellurium NPs in *P. aeruginosa* [168]. Similar results were obtained by Maruthupandy et al. by using nickel oxide NPs [169]. Zinc and titanium oxide have been documented to have strong antibacterial activity against Gram-negative and -positive bacteria, affecting their adhesion on prosthetic scaffolds [170,171]. Khan et al. obtained two morphologically different sol–gel-fabricated ZnO nanospikes with inhibitory effects on quorum sensing and BF formation in *P. aeruginosa* [172]. Specifically, ZnO nanospikes obtained from 6-week separate incubation periods exhibited the highest effect on *P. aeruginosa* virulence factors, without affecting bacterial growth. Conversely, titanium dioxide NPs were observed to affect QS only when complexed with silver [173].

### 4.3. Quorum Quenching Enzymes

Another strategy to interrupt cell-to-cell communication is the removal of signalling molecules from the environment. In this context, several studies focused on the effect of several molecules on QS pathways and quorum quenching (QQ) possibilities in several bacterial model systems [174,175,176]. The discovery of AHL antagonists able to interfere with bacterial QS signalling and induce the accelerated degradation of the AHL-dependent transcription factors attracted many researchers [177]. Several bacterial isolates that can degrade AHL by hydrolyzing the lactone bond (acyl-homoserine lactonase) and the amide linkage (AHL-acylase) have been identified [178,179,180]. Most enzyme-based QS inhibition systems involve applications against BF-producing strains of *P. aeruginosa* [181]. Recently, Packiavathy et al. reported for the first time AHL-lactonase-mediated QQ activity from marine sediment bacteria *Psychrobacter* sp. [182]. To provide evidence of the specificity in QQ enzymes, Rémy et al. investigated the activity of two lactonases targeting the signal molecules N-(3-oxododecanoyl)-L-homoserine lactone and butyryl-homoserine lactone in *P. aeruginosa* PA14 [183]. They observed a similar decreasing effect of AHL concentrations and QS gene expression associated with them. On the other hand, only the lactonase with lower efficacy on butyryl-homoserine lactone was able to inhibit *P. aeruginosa*’s pathogenicity [183]. Khalid et al. identified several bacterial strains with QQ activity and subsequently tested them against an MDR *P. aeruginosa* [184]. Their findings suggest that QQ bacterial strains and their products could be a strategy to neutralize pathogenic BF formation. To significantly increase the lactonase activity for reducing EPS and BFs and altering cell surface hydrophobicity, biofunctionalization approaches using silver and gold have been evaluated against MDR *K. pneumoniae* and *Proteus* species [185,186]. Gupta et al. indicated good activity from the silver-coated lactonase without side effects on tissue cells, suggesting it is a suitable template for designing novel anti-BF drugs [185]. Similar results have been obtained by Vinoj et al. against BFs from *Proteus* species [186]. As for the other AHL-degrading enzyme, that is acylase, similar results were obtained [187,188,189]. As described above, nanohybrid strategies based on acylase enzymes and metal NPs, graphene, or antibiotics have also been suggested for obtaining a system with enhanced antibacterial and anti-BF activities. Ivanova et al. obtained silver NPs decorated by the layer-by-layer coating of amino-cellulose and acylase able to inhibit QS-regulated virulence factors from *Chromobacterium violaceum* and BF formation from *P. aeruginosa* [190]. The same research groups obtained an enhanced antibacterial effect of gentamicin with a synergistic effect on the BF due to the combination of the antibiotic with acylase [27]. Finally, other authors describe nanoparticle systems based on graphene oxide or polyurethane with acylase to obtain inhibitory action on BF formation and to mitigate the membrane’s biofouling [191,192]. Some antibodies can interfere in bacteria cell-to-cell signalling, in addition to being biocompatible and very efficient. Marin et al. firstly report antibody-based QS-inhibition, due to the hydrolysis of N-(3-oxo-acyl) homoserine lactone mediated by monoclonal antibodies, indicating XYD-11G2 as the most efficient for inhibiting QS in *P. aeruginosa* [193]. Similarly, Kaufmann et al. suggested an immuno-pharmacotherapeutic approach against *P. aeruginosa* infections by using the monoclonal antibody RS2-1G9 [194].

## 5. Anti-BF Strategies Acting on EPS

**Figure 5:**
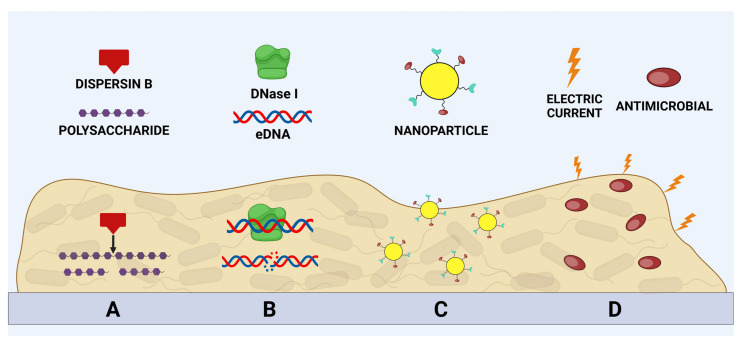
A representation of the main anti-BF strategies applied to the components of the EPS matrix. The lytic action of dispersin B on polysaccharides (**A**), the degradation of eDNA by DNase I (**B**), the use of nanoparticles functionalized with DNase and antimicrobial agents (**C**), and finally, the application of electric currents combined with molecules with antimicrobial action (**D**) are highlighted.

As previously described, the structure of the extracellular polymeric substance (EPS) allows for bacteria to protect themselves from dehydration, antibiotics, and drug penetration at bactericidal concentrations [24]. Strategies that aim to target the integrity and components of the EPS represent a promising anti-BF technique as affecting the integrity of the EPS matrix leads to the degradation of the BF [105]. The fundamental components of the matrix are proteins, polysaccharides, extracellular nucleic acids (eDNA), and some membrane vesicles. These components can act as targets to prevent the formation of the BF or for its destruction [24]. For example, anti-BF substances could prevent the polymerization and therefore the functionalization of proteins, preventing the formation of the BF itself [47]. Polysaccharides could also be used as targets for the inhibition of BF production [60]. For example, Psl and Pel in *P. aeruginosa* inhibit BF formation in *S. aureus* [31]. Conversely, in *S. aureus* protein-A inhibits Psl in *P. aeruginosa* and consequently the formation of the BF by the latter microorganism [195]. The main strategies that affect the integrity of the components of the extracellular polymeric substance will be described below (Figure 5).

### 5.1. Enzymes That Act on EPS Components

Different types of enzymes act on EPS components which lead to the disruption of the BF [105]. However, it is necessary to point out that various enzymes act as virulence factors, as they allow for the components of the EPS matrix to be degraded to promote bacterial dispersion (the last stage of the life cycle of BF formation) [196]. Dispersin B (DspB) is a protein responsible for the degradation of the BF of *Actinobacillus pleuropneumoniea* [197]. This allows for the dispersion of bacterial cells that can adhere to new nearby surfaces and therefore lead to an extension of the BF. Recombinant DspB is capable of destroying mature *S. epidermidis* BFs even at low concentrations. This characteristic is because DspB can specifically disrupt poly-*N*-acetylglucosamine (PNAG) which is one of the main polysaccharides of the BF of *S. epidermidis* [198,199]. Chen and Lee demonstrated how the combination of DspB with silver-binding peptide leads to the destruction of the matrix, and thanks to the production of AgNPs in situ, the dispersed cells are killed [200]. These authors demonstrate that although DspB is a virulence factor, with appropriate modifications, it can be used as an anti-BF strategy. Lefebvre et al. have demonstrated that proteases combined with ethylenediamine tetra-acetic acid (EDTA) can destabilize the BF and have been used for the eradication of *S. aureus* and *P. aeruginosa* BFs in patients with chronic wounds [201]. Nucleases can be considered an anti-BF [202,203]. Deoxyribonuclease I (DNase I) can degrade eDNA, causing a chain reaction that leads to a decrease in EPS matrix biomass, and as a result, makes the BF less resistant to any antibiotics [204]. Based on the same principle, Rubini et al. demonstrated that the combination of DNase with essential oils (EOs) leads to a reduction in the EPS by 85% [205]. Instead, according to Powell et al., the use of alginate oligosaccharide (OligoG) inhibits BF formation by causing an alteration in the EPS [206].

### 5.2. EPS Disruption Mediated by Nanoparticles

The use of enzymes as an anti-BF strategy is effective, but their use is limited by the high costs involved and the possible instability of the enzymes themselves [207]. In addition, mature BF makes it difficult to reach the deeper layers of the matrix. Consequently, systems capable of combining enzymes, antimicrobial agents, and nanoparticles have been designed to facilitate the dispersion of EPs and the destruction of cells in the deeper layers and to also prevent new colonization [196]. It is well known that nanoparticles can be used as anti-BF, mainly thanks to the electrostatic interactions between the NPs and the components of the EPS matrix [24]. NO-releasing silica NPs demonstrated the capacity to kill BF-based microbial cells, demonstrating how the use of nanoparticles for delivering is a promising strategy as an antimicrobial agent to microbial BFs [106]. When incorporated with silver NPs into alginate hydrogel, NO can be used for topical antibacterial applications with promising results for local applications in the combat of bacterial infections [208]. Different types of NPs have been combined with DNase and antimicrobial agents [24]. Tan et al. have effectively eradicated *S. aureus* mature BF (24 and 48 h-old) thanks to the use of positively charged chitosan NPs co-encapsulating oxacillin and DNase I. Furthermore, this system did not present cytotoxicity in the HaCat cell line (human immortalized keratinocytes) [209]. Several authors have used co-immobilized DNase I and cellobiose dehydrogenase in chitosan NPs in *Candida albicans* and *S. aureus* BFs obtaining excellent results in terms of BF destruction [210,211]. Meanwhile, Liu et al. designed MOF/Ce-based nanozymes with deoxyribonuclease (DNase) and peroxidase mimetic activities. This system can prevent bacteria from recolonizing thanks to the peroxidase-like activity of MOFs and the ability of cerium (IV) complexes to hydrolyze eDNA and disrupt established BFs [212].

### 5.3. Electrochemical Method to Deteriorate EPS

The “bioelectric effect” indicates the combination of low doses of antibiotic in a weak electric field, with the aim of disintegrating the mature biofilm [105]. It is possible to stimulate the detachment of the biofilm from a conductive surface through the application of a direct current [213]. The antibacterial activity of the electric current can be traced back to the production of toxic substances (for example, H_2_O_2_ and oxidizing radicals) following electrolysis but also to membrane damage with the consequent loss of cytoplasmic constituents [105,214]. These effects contribute to improving the minimum inhibitory concentration level leading to increased antibiotic sensitivity among BF and drug-resistant bacteria [215,216,217]. Antimicrobial agents under the influence of the electric field alter the permeability of the EPS matrix, causing the leakage of biocidal ions. The influx of those biocide ions destroys the bacterial cells through electrophoresis and electro-osmosis [218,219]. Blenkinsopp et al. have shown that this effect is not obtained with the sole application of electric current, in the absence of antimicrobial agents [220]. The antimicrobial effect related to electrical current also depends on the voltage during the electrical stimulation, as it affects the membrane potential and electrophysiology [221,222]. Alternating current (AC) or direct current (DC), or both, help implement the effect of antibiotics even at low doses [219,223,224]. Even the use of low temperature plasma, under low current, influences cell adhesion, as it decreases the EPS intensity surrounding the bacterial cells [225].

## 6. Anti-BF Strategies Mediated by Phage

**Figure 6:**
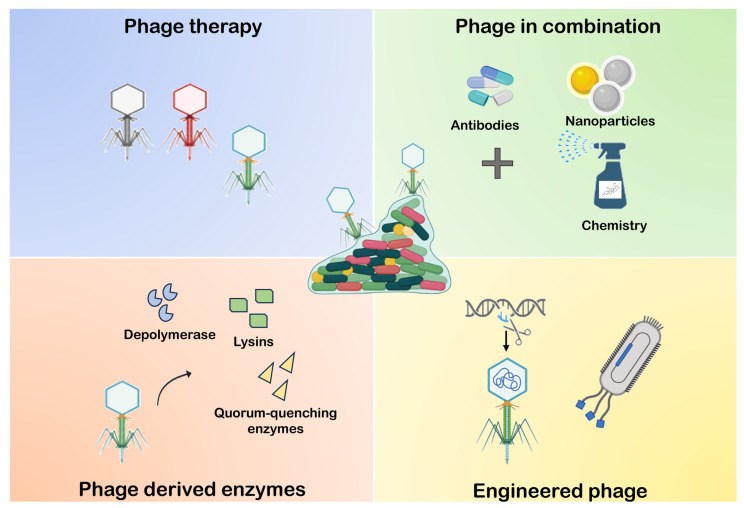
A simplified image of phage anti-BF strategies: (1) Phage therapy: based on the use of a single or cocktail phage. (2) Phage-derived enzymes, such as depolymerases, lysins, or quorum quenching enzymes are used for the dispersion of the BF matrix. (3) Phage in combination therapy: using both phages and other antimicrobial compounds, such as antibiotics, nanoparticles, or chemical disinfectants. (4) Engineered phages: the genetical modification of phages to amplify the host–species interaction range or the phage efficiency.

Bacteriophages, or simply phages, are viruses that infect bacteria and are host-dependent during self-replication [226]. Each phage has a receptor-binding protein positioned on the tail fibre, which confers specificity for a selective bacteria host [227]. Recently, with the increase in AMR, the research focus has gradually made a comeback to develop phage-based treatments able to combat pathogenic bacteria infection and also BF formation [226]. In addition, since phages are natural killers of bacteria, they represent an excellent therapeutic agent not only in clinical applications but also in other areas, such as agriculture, food control, or industry, due to their specificity and ecological safety. The antibacterial activity of phages is carried out by depolymerase and lysins, responsible for degrading capsular polysaccharides and peptidoglycan in bacterial cells, respectively [228,229,230]. Phages can be applied to prevent BF formation or to destroy existing BFs. This last strategy can be classified into the following: (i) phage therapy, based on the intra- to extracellular degradation of the bacterial cell (using a single or cocktail of lytic phages); (ii) phage-derived enzyme based on the extra- to intracellular degradation of the bacterial matrix (using lysins and/or depolymerases); (iii) the combination of phages with other antimicrobial biotic or abiotic elements; (iv) the genetic modification of phage structure or genome. In this paragraph, we focus on the four ways of phage-mediated BF remotion (Figure 6).

### 6.1. Phage Therapy

During the intra- to extracellular degradation, a phage, in the first, uses the depolymerases, present on the viral tail structure, to penetrate the BF matrix. At this point, the phage interacts with bacteria hosts leading to viral infection by genome injection [231]. The formation of lytic progenies is accompanied by the activation of holins and endolysins, responsible for piercing the cytoplasmic membrane and the degradation of bacterial peptidoglycans [232]. The use of the entire phage structure, single or in a cocktail mix, is defined as phage therapy and has shown to be effective in eradicating bacterial BF exploiting the natural ability of the phage to kill bacteria [233]. Morris et al. demonstrated a 3.3-fold reduction in BF biomass caused by *S. aureus* on three-dimensional-printed titanium after 48 h of exposure to the StaPhage cocktail, based on the combination of five *S. aureus*-specific bacteriophages [234]. On the other hand, phages in a gel-like matrix have been coated on the catheter to reduce planktonic forms and BFs of 50 tested uropathogenic *P. mirabilis* strains found on the surface [235]. Moreover, phages PSTCR4 and PSTCR6, as part of 17 characterized novel phages, exhibited an efficient reduction in well-established *P. stuartii* BFs formed in catheter models [236]. Phages, isolated from human saliva samples, showed the effective prevention and reduction in the existing BF of *S. mutans* in cariogenic dentin models, such as a decrease of up to 97% in the expression of genes involved in BF production [237,238]. Manoharadas et al. used the combination of Φ44AHJD and ΦX174 phages to disrupt the hybrid BF of *S. aureus* and *E. coli* after 72 h of incubation [239]. They also demonstrated that the use of a single phage to the mixed *E. coli*–*S. aureus*, instead, promoted the formation of the BF by the alternate strain that was not affected by the phage. A recent study showed that a phage cocktail based on four lytic phages inhibited the growth of MDR *E. coli* and caused a strong biomass reduction in the BF up to nearly 87% [240,241].

### 6.2. Phage-Derived Enzyme

As mentioned above, the enzymes depolymerases and lyases are used by phages to dissolve the BF matrix and to cleave bacterial cell walls causing the release of viral progenies, respectively. However, depolymerases are typically encoded as part of the phage structure and as such can be used as tail spike protein (TSP) or as free enzymes in the treatment of BF formation. Lysins are generally produced at the end of the phage lytic replication cycle, and in the context of BF degradation, can also be used as free enzymes [242]. For example, the depolymerase Dpo42, extracted from vB_EcoM_ECOO78 *E. coli* phage, showed the efficient degradation of the *E. coli*’s capsular polysaccharides (CPS) as well as the prevention of *E. coli* BF formation [243]. Gutiérrez et al. demonstrated the ability to inhibit and also disperse over 90% of BFs generated by different strains of *S. epidermidis* and *S. aureus* when using EPS depolymerase Dpo7, derived from bacteriophage vB_SepiS-phiIPLA7 [244]. In another study, recombinant TSP Dep42, from phage SH-KP152226, showed specific enzymatic activities against the K47 capsule of *K. pneumoniae* leading to the inhibition or degradation of its BFs. The study also showed that the combination of Dep42 with antibiotics could enhance polymyxin activity against *K. pneumoniae* BFs [245]. Recently, Shahed-Al-Mahmud et al. used φAB6 TSP to treat *A. baumannii*-adhered catheters and observed significantly fewer bacteria cells after 4 h of treatment. In an in vivo test, it was demonstrated that after 4 days, φAB6 TSP-treated zebrafish presented significantly higher survival rates compared to those without TSP treatment, suggesting the use of the treatment against MDR *A. baumannii* infections in the near future [246]. On the other hand, the use of lysin CF-301 as an anti-BF agent removed BFs from *S. aureus* or mixed-species on several surfaces, such as polystyrene, glass, surgical mesh, and catheters, with an improvement in anti-BF activity when combined with cell wall hydrolase lysostaphin [247]. Similarly, endolysin LysCSA13 showed high efficacy in removing about 80–90% of staphylococcal BF biomass on various surfaces [248]. Moreover, Yuan et al. showed the broad spectrum of antimicrobial activity of endolysin Abtn-4, isolated from *A. baumannii* phage D2, against MDR *S. aureus*, *P. aeruginosa*, *K. pneumoniae*, *Enterococcus*, and *Salmonella*, which in turn also resulted in being able to reduce formed BFs [249]. Chimeric lysin ClyH or ClyF has been found to reduce a large percentage of the BF mass of MRSA strains [250]. Recently, Vasina et al. found four endolysins LysAm24, LysAp22, LysECD7, and LysSi3 with high antibacterial activity against Gram-negative bacteria both in vitro and in vivo [251].

### 6.3. Phage Combination with Other Elements

Recent studies suggest that coupling phages with antibiotics or nanomaterials with antibacterial activity displays either the synergy or facilitation of BF treatment [29]. For example, the combination of phages with ciprofloxacin showed a synergistic effect, killing >6 log CFUs/g of fibrin clots within 6 h, in 64% of treated rats with experimental endocarditis caused by *P. aeruginosa* [252]. Similarly, a phage treatment before vancomycin or cefazolin exposure was more effective at eliminating *S. aureus* BF-associated cells. Probably, the high phage density led to the destruction of the BF matrix, then the antibiotic therapy was more efficient [253]. Recently, Cano et al. observed a biomass reduction in BF-associated prosthetic knee infection, after in vivo treatment with KpJH46Φ2 phage in combination with minocycline [254]. Stachler et al. demonstrated a potential synergistic effect between phages and chemical disinfection, such as sodium hypochlorite and benzalkonium chloride, in the remotion of BFs based on pathogen *P. aeruginosa* on the surfaces and to prevent the regeneration of dry BFs at the same time [255]. Recently, in some studies, phages were coupled with nanomaterials through physical adsorption to develop innovative alternatives for eradicating pathogenic BFs [256]. In another study, *Podoviridae* phages functionalized on magnetic nanoparticles removed about 95% of the multispecies BF (i.e., *E. coli* and *P. aeruginosa*) after 6 h of treatment [257].

### 6.4. Phage Engineering

Natural phage therapy is linked to the narrow host range and specificity [258]. However, phages can be modified by genetic engineering techniques to extend the host specificity and increase BF degradation for much broader applications [259]. Lu and Collins used engineered T7 phages to express dispersin B making it able to attack bacterial cells and facilitate the degradation of the EPS of the *E. coli* BF, resulting in a significant reduction of about 4.5 orders of magnitude of bacteria and 99% of BF mass [260]. A T7 bacteriophage was also engineered to encode a lactonase enzyme (AiiA), which has broad-range activity for the quenching of quorum sensing necessary for BF formation. The addition of this engineered T7 phage to mixed-species BFs based on *P. aeruginosa* and *E. coli* resulted in the inhibition of BF formation [261]. Moreover, Born et al. inserted the depolymerase *dpoL1* gene into the genome of phage Y2, which led to enhanced bacterial killing and had a positive effect on the dispersion of the *E. amylovora* BF [262]. Phage efficacy has also been enhanced by genetic mutation leading to the conversion from the lysogenic to the lytic phage cycle. This change enabled the successful treatment of disseminated drug-resistant *M. abscessus* infection [263]. A recent study demonstrated that the recombinant receptor of the T4-like phage conferred to the engineered phage the ability to lyse four additional hosts compared to wild-type phages, allowing for a significant inhibitory effect on mixed *E. coli* [264]. Moreover, the phage display technique, developed in 1985 by George P. Smith [265], has been applied to find peptides with the ability to degrade BFs. Phage display is based on the expression of foreign peptides on phage capsid proteins. Starting from a pool of engineered phage particles, each one with a different random peptide exposed on its capsid, the selection of an engineered phage that selectively binds to a target is obtained by biopanning cycles. This process consists of the immobilization of the target on a surface to expose it to phage peptide libraries. Then, phages that did not bind efficiently to the target are washed, while strongly phage-binding targets are eluted by different methods. The phage display technique has been used to find peptides able to detect enzymes, whole eukaryotic and prokaryotic cells, including MDR strains, and assess if they presented microbicidal activities [266,267,268,269]. In addition, it has been observed that foreign peptides can be modified in their conformation on the phage surfaces and resistance to chemical–physical environment compared to the wild-type [270,271]. These findings expand the use of engineered phages as bio-probes for medical applications. In the future the ability to specifically recognize and interact with bacteria targets could be used to deliver any antibacterial and/or anti-BF agents.

## 7. Conclusions

Biofilm formation and the increase in bacterial antibiotic resistance are causing considerable concern in the scientific community. Although there are many anti-BF strategies, we believe that new alternatives are needed so that, especially when referring to human health, we can find effective solutions, which are easy to apply and easily replicable. There is the possibility of destroying mature BFs, but this strategy seems to be the least applicable, as it could lead to dispersion and therefore to an aggravation of the problem. The possibility of preventing BF formation could represent the best strategy to stop bacteria from creating an environment favourable to their proliferation, protected from external agents and capable of resisting antibiotics. New anti-BF strategies could overcome the now widespread and inexorable resistance to antibiotics. For example, in a hospital environment, materials capable of avoiding BF formation and bacterial proliferation can not only improve healing but also avoid potential chronic or even fatal infections, considerably reducing recovery times and the, in some ways excessive, use of antibiotics.

## Data Availability

Not applicable.
